# Predictors of Long-Term Survival of Thoracoscopic Lobectomy for Stage IA Non-Small Cell Lung Cancer: A Large Retrospective Cohort Study

**DOI:** 10.3390/cancers15153877

**Published:** 2023-07-30

**Authors:** Piotr Gabryel, Piotr Skrzypczak, Alessio Campisi, Mariusz Kasprzyk, Magdalena Roszak, Cezary Piwkowski

**Affiliations:** 1 Department of Thoracic Surgery, Poznan University of Medical Sciences, Szamarzewskiego 62 Street, 60-569 Poznan, Poland; piotr.j.skrzypczak@gmail.com (P.S.); mariuszkasprzyk@ump.edu.pl (M.K.); cpiwkow@ump.edu.pl (C.P.); 2 Department of Thoracic Surgery, University and Hospital Trust—Ospedale Borgo Trento, Piazzale Aristide Stefani 1, 37126 Verona, Italy; alessio.campisi@studio.unibo.it; 3 Department of Computer Science and Statistics, Poznan University of Medical Sciences, Rokietnicka 7 Street, 60-806 Poznan, Poland; mmr@ump.edu.pl

**Keywords:** lung cancer, surgery, thoracoscopy, VATS, minimally invasive surgery, lobectomy, lymphadenectomy, mediastinal lymph node dissection

## Abstract

**Simple Summary:**

Lung cancer is a serious and, in many cases, fatal disease. If detected early, it can often be treated successfully. The best treatment results are obtained by a surgical operation which includes removing the part of the lung with the tumor and the excision of the lymph nodes from the chest. The most commonly used measure of treatment effectiveness is the five-year survival. The aim of this study was to identify factors related to 5-year survival after lung cancer surgery. We found that older age, male sex, chronic obstructive pulmonary disease and prolonged postoperative air leak were related to a lower 5-year survival rate. We also found that more accurate lymph node removal was related to a higher 5-year survival rate. These findings provide valuable insights for clinical practice and may contribute to improving the quality of treatment of early-stage NSCLC.

**Abstract:**

The standard of care for patients with early-stage non-small cell lung cancer (NSCLC) is anatomical lung resection with lymphadenectomy. This multicenter, retrospective, cohort study aimed to identify predictors of 5-year survival in patients after thoracoscopic lobectomy for stage IA NSCLC. The study included 1249 patients who underwent thoracoscopic lobectomy for stage IA NSCLC between 17 April 2007, and December 28, 2016. The 5-year survival rate equaled 77.7%. In the multivariate analysis, higher age (OR, 1.025, 95% CI: 1.002 to 1.048; *p* = 0.032), male sex (OR, 1.410, 95% CI: 1.109 to 1.793; *p* = 0.005), chronic obstructive pulmonary disease (OR, 1.346, 95% CI: 1.005 to 1.803; *p* = 0.046), prolonged postoperative air leak (OR, 2.060, 95% CI: 1.424 to 2.980; *p* < 0.001) and higher pathological stage (OR, 1.271, 95% CI: 1.048 to 1.541; *p* = 0.015) were related to the increased risk of death within 5 years after surgery. Lobe-specific mediastinal lymph node dissection (OR, 0.725, 95% CI: 0.548 to 0.959; *p* = 0.024) was related to the decreased risk of death within 5 years after surgery. These findings provide valuable insights for clinical practice and may contribute to improving the quality of treatment of early-stage NSCLC.

## 1. Introduction

According to the World Health Organization data, primary lung cancer is currently one of the most common malignancies in the world [1]. It is usually diagnosed at an advanced stage, and in most cases, it is not amenable for surgical treatment [2]. For this reason, lung cancer is the most common cause of cancer-related death in both women and men [3].

In recent years, the medical and scientific communities have made many efforts to improve lung cancer detection and treatment. Some of the most important examples are the introduction of programs for the early detection of lung cancer using low-dose chest CT (LDCT) and the development of minimally invasive surgery (MIS).

Clinical trials demonstrated that screening with LDCT reduced the risk of dying from lung cancer [4,5]. This was attributed mainly to the increased detection rate of early-stage NSCLC, which in many patients was amenable to surgical treatment [6]. In addition, LDCT frequently revealed significant incidental findings, like emphysema and coronary artery calcifications, the hallmarks of chronic obstructive pulmonary disease (COPD) and coronary artery disease [5]. Interventions targeting COPD and coronary artery disease detected on LDCT, such as smoking cessation, rehabilitation programs, pharmacological treatment and invasive procedures, may also improve long-term outcomes in these patients [7,8]. The introduction of large-scale screening programs aimed at high-risk populations and early detection of NSCLC may increase the number of patients treated with minimally invasive surgery and improve long-term treatment outcomes [9,10].

The standard of care for most patients with operable non-small cell lung cancer (NSCLC) is anatomical lung resection with mediastinal lymph node dissection (MLND) [11]. Several studies demonstrated that MIS offers superior results to open approaches for the majority of operated patients. Video-assisted thoracoscopic surgery (VATS) lobectomy has been shown to be associated with a lower incidence of postoperative complications, shorter drainage and hospitalization times, and lower postoperative pain scores compared to open lobectomy [12,13]. Studies indicate that VATS lobectomy in NSCLC may be associated with lower 30- and 90-day mortality [14] and improved long-term survival [15]. Robotic-assisted thoracic surgery is a relatively new but rapidly developing approach for NSCLC. Studies demonstrated that robotic surgery has all the advantages of MIS compared to open surgery [16]. Recent research indicates that robotic surgery for lobectomy and segmentectomy may be associated with less blood loss, lower conversion rate, more thorough lymph node dissection, lower complication rate, shorter duration of chest drainage and hospital stay, and lower recurrence rate compared to VATS [17]. The disadvantage of robotic surgery is the cost of the procedure, far exceeding VATS, which will undoubtedly limit the development of robotic surgery in low- and middle-income countries [18]. For this reason, VATS will likely remain the approach used for anatomical lung resections in many parts of the world in the foreseeable future.

For a long time, lobectomy was considered the standard of care for early-stage NSCLC [19]. Recently published results of JCOG0802 [20] and CALGB 140503 [21] trials indicated favorable short-term and long-term results of segmentectomy, compared to lobectomy, for peripheral IA1 and IA2 NSCLC. However, there are many knowledge gaps and potential pitfalls associated with segmentectomy. Segmentectomy itself and lymphadenectomy during the procedure are difficult. Failure to obtain a sufficient margin of the lung parenchyma and inaccurate lymphadenectomy may result in incomplete resection, non-detection of nodal metastases and increased local recurrence rate [22]. Moreover, many patients with stage IA NSCLC will not be amenable to segmentectomy due to the size and location of the nodule, and for this group VATS lobectomy will still be the procedure of choice.

Long-term survival is the most important indicator of the quality of lung cancer diagnostics and oncological treatment. The outcomes of NSCLC treatment depend primarily on the cancer stage according to the TNM classification [11]. Several other clinical, surgical and histopathological indicators, such as age, sex, comorbidities, extent of resection, neoadjuvant therapy, nodal status and completeness of resection were found to influence survival for stages I to IV NSCLC [23]. Icard et al. demonstrated that pre-surgery weight loss, sarcopenia and lower Body Mass Index negatively influenced the long-term outcomes of surgery for all stages of NSCLC [24]. Other factors that are currently being investigated for prognostic significance in localized NSCLC include circulating tumor DNA, epigenetic alterations and tumor molecular alterations, such as epidermal growth factor receptor mutations and tumor suppressor genes mutations [25]. However, predictors of the outcomes of minimally invasive surgery for the earliest stages of lung cancer are poorly studied. The aim of the study was to identify factors related to long-term survival in patients after VATS lobectomy for early-stage NSCLC.

## 2. Materials and Methods

The requirements for the ethics approval and for the patients’ consent to collect, analyze and publish anonymized data of this retrospective study were waived by the Poznan University of Medical Sciences Bioethics Committee.

### 2.1. Healthcare Setting, Data Source, Definitions

In Poland, approximately 20,000 new cases of lung cancer are diagnosed annually [26]. Nearly 20% of NSCLC patients are treated surgically. Surgical treatment is provided by 29 thoracic surgery departments. Lung cancer resections can only be performed by board-certified thoracic surgeons and thoracic surgery residents under supervision of thoracic surgeons. Although there is a well-developed private healthcare sector in Poland, lung cancer operations are performed exclusively within the public healthcare system. All data on surgical treatment of lung cancer in Poland are collected by the Polish Lung Cancer Study Group Database. The database is maintained by the National Institute of Tuberculosis and Lung Diseases in Warsaw. Entering the data into the database is mandatory and is the responsibility of the surgeons from individual departments. The database includes data on demographic and epidemiologic characteristics, pulmonary function tests, radiological examinations, invasive diagnostic procedures, date and type of surgery, lymph node stations and number of lymph nodes removed for each station, postoperative care, including chest tube duration, complications and date of discharge, histology, clinical and pathological staging, and follow-up. Data on the date of death is updated according to the official national records. Histological type and outcomes of surgery are defined in the database in accordance with the WHO classification [27] and European Society of Thoracic Surgery definitions [28]. The NSCLC stage of all patients included in the database is regularly updated to the latest TNM classification. In this study, the eighth edition of the TNM classification was used [11]. Systematic nodal dissection (SMLND) and lobe-specific (L-SMLND) were defined according to the IASLC guidelines [29]. SMLND included all nodal stations on the operated side. Depending on the type of lobectomy, the L-SMLND included the following nodal stations:

1. Right upper and right middle lobectomy: right upper paratracheal (2R), right lower paratracheal (4R) and subcarinal (7) nodes.
2. Right lower lobectomy: right lower paratracheal (4R), subcarinal (7), and paraesophageal (8) or pulmonary ligament (9) nodes.
3. Left upper lobectomy: aorto-pulmonary window (5), paraaortic (6) and subcarinal (7) nodes.
4. Left lower lobectomy: subcarinal (7), paraesophageal (8) and pulmonary ligament (9) nodes.

### 2.2. Study Design

This multicenter, retrospective, cohort study included patients who underwent VATS lobectomy for pathologic stage IA non-small cell lung cancer between 17 April 2007, and 28 December 2016. All patients were followed up to the date of death or for at least 5 years after the surgery. The data were retrieved from the Polish Lung Cancer Study Group Database on 13 November 2022. The exclusion criteria were as follows: surgical approach other than VATS (thoracotomy, sternotomy and robotic-assisted surgery), sublobar resection, extended resection (with large vessels, chest wall, diaphragm and pericardium, and bronchial and/or vascular sleeve resections), histology other than NSCLC (metastasis, small-cell lung cancer and benign lesions), neoadjuvant therapy (radio-, chemo- or immunotherapy) and lack of information on surgical approach. None of the patients received postoperative chemotherapy, targeted treatment or immunotherapy. The primary study endpoint was the five-year survival rate. In this study, we assessed the impact of pre-, intra- and postoperative factors on five-year survival. 

### 2.3. Statistical Analyses

The numerical data are presented as median (interquartile range), while categorical as number (percentage). We performed the Cox proportional-hazards model. The dependent variable was 5-year survival. Initially, we performed univariate analysis using all variables describing the study—all presented in Table 1. The results were considered statistically significant at the *p* value < 0.05. The relationships between individual variables were analyzed using the Spearman’s coefficient. Part of the data that was strongly correlated with each other was excluded from the multivariate model. The multivariate Cox proportional hazards survival regression included data that were significant in the univariate analysis. Kaplan–Meier curves were also prepared for dichotomous variables. Statistical analyses were conducted using IBM^®^ SPSS^®^ Statistics v. 27th (PS Imago Pro 8).

## 3. Results

The study included 1249 patients after VATS lobectomy for pathologic stage IA non-small cell lung cancer who met all study criteria. The median follow-up time from the date of surgical operation to the date of the data collection was 90.4 months (range 174.1 to 70.5 months). The 5-year survival rate of the whole cohort was 77.7%. Five-year survival for stages IA1, IA2 and IA3 amounted to 83.8%, 79.5% and 73.9%, respectively.

Univariate analysis showed that the factors associated with 5-year survival were as follows: age (*p* < 0.001), sex (*p* < 0.001), comorbidities (*p* = 0.002), COPD (*p* < 0.001), Charlson Comorbidity Index (*p* < 0.001), FEV1% (*p* < 0.001), FVC% (*p* < 0.001), clinical stage (*p* = 0.006), L-SMLND (*p* = 0.015), pathological stage (IA1 vs. IA2 vs. IA3) (*p* = 0.006), chest tube duration (*p* = 0.011), prolonged air leak (*p* < 0.001), residual air space (*p* = 0.047) and supraventricular arrythmia (*p* = 0.035). The data on the baseline, surgical, postoperative and histopathological characteristics and the results of univariate analyses are shown in Table 1 and Table 2.

There was a significant correlation between COPD and FEV1 (R = −0.330, *p* < 0.001), COPD and FVC (R = −0,21, *p* < 0.001), and FEV1 and FVC (R = 0.75, *p* < 0.001) (Supplementary Table S1). Because the differences in FEV1 and FVC values between the groups were small and difficult to interpret in clinical practice (Table 1), COPD was included in the multivariate analysis. Similarly, clinical and pathological staging were correlated (R = 0.106, *p* < 0.001). Pathological stage was included in the multivariate analysis because it is a more accurate measure of the actual NSCLC stage. We also did not include the overall comorbidity rate in the model because of the comorbidities; only COPD was significantly associated with five-year survival, and the overall burden of comorbidities in the multivariate model was analyzed as the Charlson Comorbidity Index. In addition, since the duration of pulmonary drainage resulted almost exclusively from prolonged air leakage, the latter variable was included in the model. The other variables with a *p*-value below 0.05 in the univariate analysis were included in the multivariate Cox proportional hazards model.

The variables that were significant in the multivariate Cox proportional hazards survival regression were age (OR, 1.025, 95% CI: 1.002 to 1.048; *p* = 0.032), sex (OR, 1.410, 95% CI: 1.109 to 1.793; *p* = 0.005), COPD (OR, 1.346, 95% CI: 1.005 to 1.803; *p* = 0.046), prolonged air leak (OR, 2.060, 95% CI: 1.424 to 2.980; *p* < 0.001), pathological stage (OR, 1.271, 95% CI: 1.048 to 1.541; *p* = 0.015) and L-SMLND (OR, 0.725, 95% CI: 0.548 to 0.959; *p* = 0.024). Older age, male sex, higher pathological stage (IA1 vs. IA2 vs. IA3), presence of COPD and occurrence of prolonged air leak increased the risk of death within 5 years. Patients for whom lymph nodes assessment met the IASLC criteria for L-SMLND had a lower risk of death. The results of the multivariate analysis are presented in Table 3.

**Figure 1:**
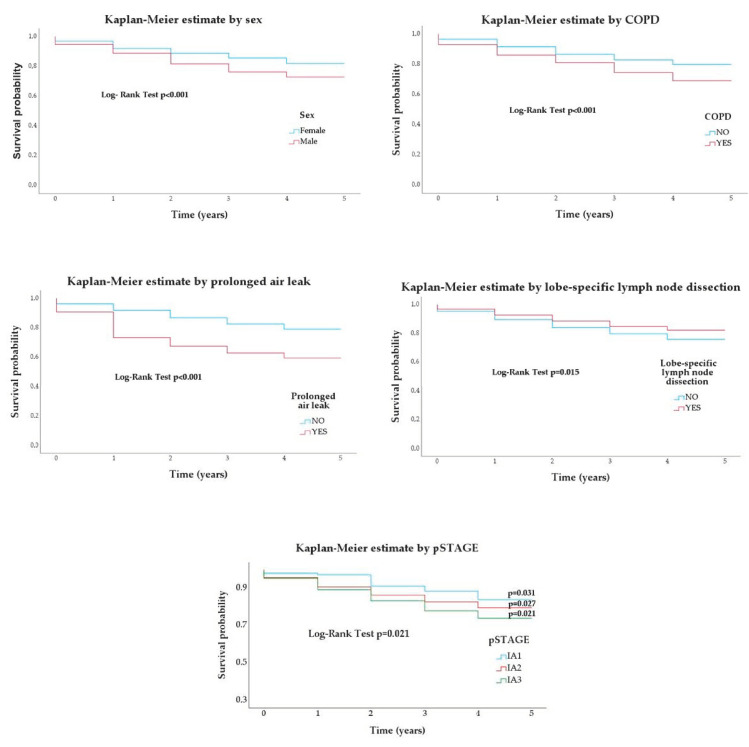
Kaplan-Meier survival estimates by sex, chronic obstructive pulmonary disease (COPD), prolonged air leak, lobe-specific lymph node dissection and pathological stage (pSTAGE).

Kaplan–Meier survival curves for sex, COPD, prolonged air leak, L-SMLND and pathological stages are plotted in Figure 1.

## 4. Discussion

The most important prognostic factor for the long-term outcomes in the whole group of patients operated for NSCLC is the TNM stage, including the size and invasion of the tumor, metastases to the lymph nodes and distant metastases [11]. Patients with stage IA NSCLC have a relatively low risk of recurrence and generally have a favorable prognosis. For this reason, factors other than the stage of the disease may be of relatively greater importance for the prognosis of survival in this group. This study identified certain variables associated with 5-year survival in patients after VATS lobectomy for stage IA NSCLC. We have shown that the prognosis was worse in elderly male patients and in the case of coexisting COPD. We also found that the quality of the lymphadenectomy may influence the long-term outcomes.

The influence of age and sex on the long-term outcomes of NSCLC treatment has been quite extensively discussed in the literature. Our study confirmed that older age and male sex were associated with the increased risk of death within 5 years after surgery. Similar results were obtained by Sigel et al., who showed that male patients over 80 years of age operated for stage I NSCLC had a worse prognosis than younger patients [30]. Sagerup et al. found that women are diagnosed with less advanced disease and that men have an increased risk of death at 5 years, irrespective of stage, age, period of diagnosis and selected histological subgroups [31]. Elderly male patients usually have more comorbidities, increased surgical risk and higher perioperative mortality [32,33]. In addition, in patients with advanced stages and with the progression of NSCLC, a lower treatment receipt rate [34] and poorer tolerance of oncological treatment related to older age may also influence survival [35]. Most likely, these factors may negatively influence the long-term outcomes of NSCLC surgery. A thorough assessment of surgical risk and appropriate qualification for surgery are crucial for achieving good long-term results in this group of patients.

Another finding of our study is a lower 5-year survival rate in patients with chronic obstructive pulmonary disease. COPD is one of the most common respiratory system diseases in adults [36]. Due to a common etiological factor—smoking—COPD is diagnosed in many patients with lung cancer [37]. Previous studies demonstrated that COPD increases the risk of postoperative complications [38] and is associated with poorer long-term outcomes of NSCLC surgery, resulting primarily from the progressive decrease in respiratory function leading to respiratory failure [39]. COPD treatment is long-term and most often does not prevent disease progression. Smoking cessation is the most important intervention for COPD [40]. In patients operated for NSCLC, smoking cessation also significantly reduces the risk of postoperative complications [41], which was the main reason for including this intervention in the enhanced recovery after surgery (ERAS) protocol [42]. Pharmacological treatment and physical therapy have the potential to impact the course of COPD, but their effect on the prognosis of patients with comorbid COPD and cancer is unknown [43]. Lung transplantation is an effective treatment for advanced COPD, but it is absolutely contraindicated for lung cancer [44]. The results of a few studies in small groups of patients demonstrated that lung transplantation in patients with coexisting undetected stage I NSCLC was associated with a low recurrence rate and generally good prognosis [45]. As already mentioned, malignant neoplastic disease is an absolute contraindication to lung transplantation nowadays. However, as organ transplantation and lung cancer diagnosis and treatment develop, lung transplantation could become an option for a highly selected group of patients with concomitant severe COPD and lung cancer.

Apart from COPD, other comorbidities and comorbidity indexes, such as Charlson Comorbidity Index (CCI) or Elixhauser Index, were also indicated as risk factors for early and late postoperative morbidity and mortality [46,47]. This study found no relationship between CCI and the outcomes of NSCLC surgery. This can be explained by the limitations on the index. First, CCI was designed to assess the prognosis of chronic diseases, not lung cancer. Secondly, advances in healthcare in recent years have caused the impact of certain diseases (for example Acquired Immunodeficiency Syndrome, AIDS) on the value of the index to be overestimated nowadays. Thirdly, several factors included in the index are correlated. In our study, CCI was correlated with age, sex, FEV1, FVC, COPD and postoperative AF (Supplementary Table S1). Further studies should focus on designing new predictive models, as well as determining the role of comorbidities and their prophylaxis and treatment in the long-term outcomes of NSCLC. Wide-scale implementation of the ERAS protocols may be particularly important for improving NSCLC treatment outcomes.

Our study showed that prolonged air leak was associated with poorer long-term outcomes of NSCLC surgery. Previous studies have demonstrated the relation between the occurrence of postoperative complications and lower overall survival, cancer-specific survival and recurrence-free survival [48]. Wang et al. found that major pulmonary complications, of which prolonged air leak was the most common, were associated with worse long-term outcome of VATS lobectomy [49]. There may be several reasons for the association between prolonged air leakage and long-term outcomes revealed in our study. Firstly, prolonged air leak can delay the recovery process and increase the risk of other postoperative complications which increase mortality, such as venous thromboembolism and pneumonia. Secondly, incomplete lung re-expansion due to prolonged air leak may lead to reduced lung volume, deterioration of lung function, worse exercise capacity and impaired rehabilitation. Thirdly, prolonged air leak may not be a direct cause of lower survival rate, but rather a manifestation of poor lung quality resulting from smoking and COPD, which are known predictors of worse outcomes of lung surgery [48,50]. Based on the currently available literature, none of the possibilities discussed above can be ruled out, and further research is needed in this area. Regardless of the mechanism of the relationship between prolonged air leak and long-term survival, measures to reduce its incidence should be introduced. Surgical techniques to prevent air leak and methods of intraoperative and postoperative management in the event of an air leak are quite well described. Firstly, the lung parenchyma and fused interlobar fissures should be dissected during surgery only with the staplers because the use of electrosurgical devices is associated with an increase in the incidence of air leak [51]. Secondly, if an intraoperative air leak is detected, the site of the leak should be treated with sutures or sealants [52]. Third, passive drainage should be used in the postoperative period since active drainage may contribute to prolonging the duration of air leak [53]. Fourthly, electronic drainage devices are related to the shorter duration of chest tube placement, shorter hospital stay and lower complications rate compared to the classic four-chamber drainage devices and should rather be used for postoperative drainage [54,55]. Finally, in the event of postoperative air leak, techniques such as an autologous blood patch may be considered for treatment [56]. Further research should focus on determining the mechanisms of the impact of prolonged air leak on the long-term outcomes of the NSCLC surgical operations and on developing the guidelines for the prevention and treatment of intraoperative and postoperative air leak.

According to the IASLC guidelines, lymphadenectomy is a prerequisite for complete surgery for lung cancer [29]. The ESTS guidelines indicate that systematic MLND should be performed and should include all nodal stations on the operated side [57]. Lobe-specific MLND is also acceptable for early-stage NSCLC [29]. This is supported by the study of Deng et al., which demonstrated that in most cIA NSCLC with postoperative pN2 features, metastases occur in lymph nodes specific for a given lobe of the lung [58]. 

The quality of lymphadenectomy affects the long-term outcomes of lung cancer treatment. Osarogiagbon et al. demonstrated that a higher number of lymph nodes removed was associated with better long-term outcomes of node-negative NSCLC treatment [59]. Lardinois et al. revealed that patients with stage I NSCLC had a significantly longer disease-free survival after SMLND than after nodal sampling [60]. Another study by Yang et al., which included patients who were followed up for at least 10 years after surgery for stage I NSCLC, found that SMLND was associated with better survival than sampling [61]. Regarding the comparison of L-SMLND and SMLND, a recently published study by Hunag et al. showed that both methods give comparable long-term outcomes in patients with stage cI NSCLC [62]. The results of our study also indicate that patients with stage I NSCLC should undergo at least L-SMLND. The reason for the better long-term results in patients with L-SMLND is probably the improved detection of lymph node metastases and more accurate lung cancer staging. Whether removing the metastatic lymph nodes improves survival is questionable and requires further research. Since the quality of MLND is often insufficient, the thoracic surgery community should take steps aimed at its improvement, such as surgical education and training, and the introduction of techniques to facilitate surgical operations, including the use of advanced electrosurgical devices and robotic surgery [16,17,18,63].

One of the issues that should be discussed in relation to the current study is pre-operative staging. In the studied cohort of patients, positron emission tomography-computed tomography (PET-CT) scanning and invasive mediastinal staging were relatively rarely performed. While the low rate of invasive mediastinal staging for cIA NSCLC is not surprising, the low rate of PET-CT raises some concerns. PET-CT is currently well-established in the preoperative diagnosis of lung cancer and allows the detection of metastases to the mediastinal lymph nodes and distant metastases [64]. More frequent use of PET-CT could improve radiologic mediastinal staging and would help guide invasive staging for suspected nodal metastases [65]. On the other hand, this study showed that the PET-CT rates did not differ between groups of patients who survived and who died within five years after surgery. This may suggest that PET-CT in early lung cancer may not have as much impact on long-term results as we expect. The low rate of PET-CT in the present study can be explained by the time frame of the study, i.e., years 2007 to 2016, in which this method was only just introduced to general use. Currently, in Poland, PET-CT is performed in most centers in over 90% of patients qualified for NSCLC surgery. The assessment of the impact of PET-CT on the long-term results of early-stage NSCLC treatment would undoubtedly be an interesting topic for further research.

### Strengths and Limitations

The main advantage of the study is that it covers the entire population of patients operated for stage IA NSCLC in one large European country. This means that, with a high probability, the results of the study could be generalized to countries with a similar level of development and comparable healthcare systems. On the other hand, the diversity of cancer biology in various geographical areas of the world and differences in the functioning of health care systems may result in differences in predictive factors of NSCLC surgery. In order to assess the generalizability of the results, it would be necessary to test this model on databases from other geographic regions. In addition, the large number of subjects, good-quality data on surgery, post-operative care and histopathology results, and long-term follow-up make the study results valuable for thoracic surgeons involved in the treatment of NSCLC.

The main limitations of the study were the lack of data such as on smoking, categorization of COPD by different stages, type of approach for VATS (uniportal vs. multiportal) and location of tumors (central versus peripheral). Information on postoperative radiotherapy was incomplete and therefore was not included in the statistical analysis. However, the incidence of incomplete resection, which is the only indication for radiotherapy in this group of patients, did not differ between the two groups, and we have no reason to believe that the groups of patients were not homogeneous in this regard. Moreover, the accuracy of data on local recurrence and cause of death (cancer-related vs. unrelated) was low, and these were not included in the analysis.

## 5. Conclusions

Long-term results of VATS lobectomy for stage IA NSCLC are worse in elderly male patients with coexisting COPD. In this group of patients, special attention should be paid to qualification and preparation for surgery, including preoperative rehabilitation and appropriate pulmonological treatment. Prolonged air leak may negatively affect five-year survival after NSCLC surgery. Aggressive use of air leak prevention and treatment methods can reduce the incidence of prolonged air leak and improve treatment outcomes. The introduction of the ERAS program, which incudes several actions that can hasten recovery in high-risk patients and reduce the incidence of air leak, may also be of value.

The quality of lymphadenectomy affects the long-term outcomes of surgical treatment of patients with early-stage NSCLC. However, intraoperative evaluation of lymph nodes is often not accurate enough. In order to improve the results of NSCLC treatment, a number of measures can be introduced, such as surgical education and training focused on MLND, introducing new techniques that facilitate surgical operations, including high-energy devices and robotic surgery, and research aimed at identifying factors affecting the quality of MLND.

The findings of this study provide valuable insights for clinical practice and may contribute to improving the outcomes of treatment of early-stage NSCLC.

## Figures and Tables

**Table 1:** Associations between the preoperative variables and the outcome at five years after VATS lobectomy for stage IA NSCLC.

	Outcome at 5 Years after Surgery		*p* Value
	Alive (*n* = 970)	Dead (*n* = 279)	
Age, mean (SD)	63.0 (SD: 8.2)	65.6 (SD: 7.6)	<0.001 *
Male, *n* (%)	433 (44.6)	161 (57.7)	<0.001 *
Comorbidities, *n* (%)	681 (70.2)	223 (79.9)	0.002 *
Arterial hypertension	415 (42.7)	135 (48.4)	0.101
Chronic obstructive pulmonary disease	179 (18.5)	80 (28.7)	<0.001 *
Diabetes mellitus	116 (12.0)	39 (14.0)	0.312
Coronary heart disease	102 (10.5)	36 (12.9)	0.270
Other neoplastic disease	84 (8.7)	26 (9.3)	0.720
Peripheral arterial disease	31 (3.2)	15 (5.4)	0.067
Heart failure	13 (1.3)	4 (1.4)	0.774
Cerebrovascular disease	12 (1.2)	4 (1.4)	0.764
Chronic kidney disease	6 (0.6)	3 (1.0)	0.413
ThRCRI, *n* (%)			0.256
Group A	858 (88.5)	239 (85.7)	
Group B	112 (11.5)	40 (14.3)	
CCI, median (IQR)	2 (IQR, 2 to 4)	3 (IQR, 2 to 4)	<0.001 *
FEV1%, *%* (SD)	90.6 (SD: 19.7)	84.7 (SD: 20.4)	<0.001 *
FVC%, *%* (SD)	102.6 (SD: 18.0)	96.6 (SD: 18.2)	<0.001 *
Patients with PET-CT	235 (24.2)	71 (25.4)	0.679
Patients with invasive mediastinal staging, *n* (%)	126 (12.9)	38 (13.6)	0.740
Clinical TNM stage, *n* (%)			0.006 *
cIA	715 (78.7)	188 (70.7)	
cIB	125 (13.8)	50 (18.8)	
cII	47 (4.9)	16 (5.7)	
cIII	22 (2.4)	12 (4.3)	
No data	61 (6.3)	13 (4.7)	

SD = standard deviation; IQR = interquartile range; ThRCRI = Thoracic Revised Cardiac Risk Index; CCI = Charlson Comorbidity Index; ppFEV1% = predicted postoperative percentage of calculated forced expiratory volume in 1 s; FVC% = predicted postoperative percentage of calculated forced vital capacity; PET-CT = positron emission tomography—computed tomography. * Statistically significant (*p* < 0.05).

**Table 2:** Associations between the surgical, postoperative and histopathological characteristics and the outcome at five years after VATS lobectomy for stage IA NSCLC.

	Outcome at 5 Years after Surgery		*p* Value
	Alive (*n* = 970)	Dead (*n* = 279)	
Side of surgery, *n* (%)			0.070
Right	559 (57.6)	143 (51.3)	
Left	411 (42.4)	136 (48.7)	
Chest tube duration, median (IQR)	3 (IQR, 2 to 4)	4 (IQR, 3 to 4)	0.011 *
Hospital stay duration, median (IQR)	6 (IQR, 5 to 8)	6 (IQR, 5 to 8)	0.701
Complications, *n* (%)	188 (19.4)	97 (34.8)	<0.001 *
Prolonged air leak	51 (5.3)	35 (12.5)	<0.001 *
Residual air space	21 (2.2)	12 (4.3)	0.047 *
Pneumonia/atelectasis	33 (3.4)	10 (3.6)	0.401
Atrial fibrillation	36 (3.7)	18 (6.5)	0.035 *
Reoperation	13 (1.3)	5 (1.8)	0.437
Bleeding or hemothorax	10 (1.0)	3 (1.1)	0.819
Psychosis	6 (0.6)	2 (0.7)	0.799
Transfusion	19 (1.9)	19 (6.8)	<0.001
Other complications	17 (1.8)	12 (4.3)	0.006
Histology, *n* (%)			0.233
Squamous cell carcinoma	233 (24.0)	73 (26.1)	
Adenocarcinoma	521 (53.7)	155 (55.6)	
Other histological type	216 (22.3)	51 (18.3)	
With clear surgical margins (R0), *n* (%)	968 (99.8)	278 (99.6)	0.894
Pathological TNM stage, *n* (%)			0.006 *
pIA1	93 (9.6)	18 (6.5)	
pIA2	518 (53.4)	134 (48.0)	
pIA3	359 (37.0)	127 (45.5)	
Patients with L-SMLND, *n* (%)	296 (30.5)	64 (22.9)	0.015 *
Patients with SMLND, *n* (%)	41 (4.2)	13 (4.6)	0.768

IQR = interquartile range; L-SMLND = lobe-specific mediastinal lymph node dissection; SMLND = systematic mediastinal lymph node dissection. * Statistically significant (*p* < 0.05).

**Table 3:** Results of the multivariate Cox proportional hazards survival regression of variables related to the all-cause 5-year death rate in patients after VATS lobectomy for stage IA NSCLC.

	Odds Ratio	95% Confidence Interval	*p* Value
Age	1.025	1.002 to 1.048	0.032 *
Sex, male	1.410	1.109 to 1.793	0.005 *
COPD	1.346	1.005 to 1.803	0.046 *
Charlson comorbidity index	1.044	0.913 to 1.195	0.528
Lobe-specific MLND	0.725	0.548 to 0.959	0.024 *
Pathological stage	1.271	1.048 to 1.541	0.015 *
Prolonged air leak	2.060	1.424 to 2.980	<0.001 *
Residual air space	1.555	0.854 to 2.833	0.149
Supraventricular arrythmia	1.494	0.921 to 2.424	0.104

COPD = chronic obstructive pulmonary disease; MLND = mediastinal lymph node dissection. * Statistically significant (*p* < 0.05).

## Data Availability

The data underlying this article will be shared on reasonable request to the corresponding author.

## Supplementary Materials

The following supporting information can be downloaded at: https://www.mdpi.com/article/10.3390/cancers15153877/s1, Table S1: Correlations between variables related to five year survival of VATS lobectomy for stage IA NSCLC.
