# Supplementary material for: Predictors of Long-Term Survival of Thoracoscopic Lobectomy for Stage IA Non-Small Cell Lung Cancer: A Large Retrospective Cohort Study
Source: Cancers (Basel). 2023 Jul 30;15(15):3877. doi: 10.3390/cancers15153877 (PMC10416904; doi:10.3390/cancers15153877)
Supplement: Supplementary file 1 [file cancers-15-03877-s001.zip › cancers-2512068-supplementary.pdf]

Table S1. Correlations between variables related to five year survival of VATS lobectomy for stage IA NSCLC.

			Age	Sex	LSMLND	RAS	AF	COPD	PAL_	pSTAGE	cSTAGE	FEV1%	FVC%	CCI
Spearman rho	Age	Correlation coefficient	1,000	,094**	-,049	-,019	,088**	,136**	,032	,045	,001	-,096**	-,098**	,744**
		Significance	.	<,001	,082	,508	,002	<,001	,265	,112	,986	,004	,004	<,001
		N	1249	1249	1249	1249	1249	1249	1249	1249	1175	879	851	1249
	Sex	Correlation coefficient	,094**	1,000	-,015	-,007	,050	,078**	,070*	,046	,075**	-,172**	-,250**	,121**
		Significance	<,001	.	,599	,806	,078	,006	,013	,101	,010	<,001	<,001	<,001
		N	1249	1249	1249	1249	1249	1249	1249	1249	1175	879	851	1249
	LSMLND	Correlation coefficient	-,049	-,015	1,000	-,017	,004	,023	-,005	,040	-,012	,017	,007	-,048
		Significance	,082	,599	.	,556	,894	,410	,846	,154	,671	,610	,838	,091
		N	1249	1249	1249	1249	1249	1249	1249	1249	1175	879	851	1249
	RAS	Correlation coefficient	-,019	-,007	-,017	1,000	-,010	-,010	,192**	-,005	-,040	-,042	-,014	,005
		Significance	,508	,806	,556	.	,712	,714	<,001	,863	,171	,212	,693	,853
		N	1249	1249	1249	1249	1249	1249	1249	1249	1175	879	851	1249
	AF	Correlation coefficient	,088**	,050	,004	-,010	1,000	,095**	-,042	-,022	,016	-,065	-,054	,109**
		Significance	,002	,078	,894	,712	.	<,001	,136	,440	,591	,055	,114	<,001
		N	1249	1249	1249	1249	1249	1249	1249	1249	1175	879	851	1249
	COPD	Correlation coefficient	,136**	,078**	,023	-,010	,095**	1,000	,071*	,032	,058*	-,330**	-,211**	,385**
		Significance	<,001	,006	,410	,714	<,001	.	,011	,256	,049	<,001	<,001	<,001
		N	1249	1249	1249	1249	1249	1249	1249	1249	1175	879	851	1249
	PAL	Correlation coefficient	,032	,070*	-,005	,192**	-,042	,071*	1,000	,041	,065*	-,115**	-,053	,024
		Significance	,265	,013	,846	<,001	,136	,011	.	,150	,027	<,001	,120	,392
		N	1249	1249	1249	1249	1249	1249	1249	1249	1175	879	851	1249
	pSTAGE	Correlation coefficient	,045	,046	,040	-,005	-,022	,032	,041	1,000	,106**	-,105**	-,077*	,050
		Significance	,112	,101	,154	,863	,440	,256	,150	.	<,001	,002	,024	,080
		N	1249	1249	1249	1249	1249	1249	1249	1249	1175	879	851	1249
	cSTAGE	Correlation coefficient	,001	,075**	-,012	-,040	,016	,058*	,065*	,106**	1,000	-,036	-,041	,053
		Significance	,986	,010	,671	,171	,591	,049	,027	<,001	.	,296	,242	,071
		N	1175	1175	1175	1175	1175	1175	1175	1175	1175	823	796	1175
	FEV1%	Correlation coefficient	-,096**	-,172**	,017	-,042	-,065	-,330**	-,115**	-,105**	-,036	1,000	,753**	-,191**
		Significance	,004	<,001	,610	,212	,055	<,001	<,001	,002	,296	.	<,001	<,001
		N	879	879	879	879	879	879	879	879	823	879	850	879
	FVC%	Correlation coefficient	-,098**	-,250**	,007	-,014	-,054	-,211**	-,053	-,077*	-,041	,753**	1,000	-,160**
		Significance	,004	<,001	,838	,693	,114	<,001	,120	,024	,242	<,001	.	<,001
		N	851	851	851	851	851	851	851	851	796	850	851	851
	CCI	Correlation coefficient	,744**	,121**	-,048	,005	,109**	,385**	,024	,050	,053	-,191**	-,160**	1,000

	Significance	<,001	<,001	,091	,853	<,001	<,001	,392	,080	,071	<,001	<,001	.
	N	1249	1249	1249	1249	1249	1249	1249	1249	1175	879	851	1249

**Correlation significant at the level of 0.01 (Two-sided); *correlation significant at the level of 0.05 (Two-sided).
